# Illinois

**Published:** 1896-09

**Authors:** 


*Illinois.* The Illinois Live-stock Commissioners, since July 23, 1894, have inspected and tagged 17,400 cattle; 12,642 cattle have been tagged with round tags, which allows them to be sold on the open market on their own merits; 4938 cattle have been tagged with square tags, which were held for post-mortem examination, as it was not certain on ante-mortem examination whether they were fit for human food. Out of the 4938 cattle held for post-mortem 1444 were passed that have sold at an average of $5.50 per hundred, dressed weight. The Commissioners have caused 3494 cattle to be tanked and rendered unfit for food in the presence of a member of the Board, a director of the Live-stock Exchange, and a member of the City Board of Health. They have saved to the shipper and producer of live-stock by their system of inspection at least $335,000, this being the amount over and above what the cattle shipped would have been sold for under the old regime, and have protected the consumer by destroying 3494 head of cattle that were badly diseased and which otherwise would have been eaten.

